# Current trends and outcomes of non-elective neurosurgical care in Central Europe during the second year of the COVID-19 pandemic

**DOI:** 10.1038/s41598-022-18426-y

**Published:** 2022-08-27

**Authors:** Ondra Petr, Lukas Grassner, Freda M. Warner, Michaela Dedeciusová, Richard Voldřich, Philipp Geiger, Konstantin Brawanski, Sina Gsellmann, Laura C. Meiners, Richard Bauer, Sascha Freigang, Michael Mokry, Alexandra Resch, Thomas Kretschmer, Tobias Rossmann, Francisco Ruiz Navarro, Harald Stefanits, Andreas Gruber, Mathias Spendel, Christoph Schwartz, Christoph Griessenauer, Franz Marhold, Camillo Sherif, Jonathan P. Wais, Karl Rössler, Jakob J. Zagata, Martin Ortler, Wolfgang Pfisterer, Manfred Mühlbauer, Felipe A. Trivik-Barrientos, Johannes Burtscher, Lukáš Krška, Radim Lipina, Martin Kerekanič, Jiří Fiedler, Petr Kasík, Vladimír Přibáň, Michal Tichý, Vladimír Beneš, Petr Krůpa, Tomáš Česák, Robert Kroupa, Andrej Callo, Pavel Haninec, Daniel Pohlodek, David Krahulík, Alena Sejkorová, Martin Sameš, Josef Dvořák, Andriana Juričeková, Pavel Buchvald, Robert Tomáš, Jan Klener, Vilém Juráň, Martin Smrčka, Petr Linzer, Miroslav Kaiser, Dušan Hrabovský, Radim Jančálek, John L. K. Kramer, Claudius Thomé, David Netuka

**Affiliations:** 1 Department of Neurosurgery, Medical University Innsbruck, Innsbruck, Austria; 2 International Collaboration on Repair Discoveries (ICORD), University of British Columbia, Vancouver, BC Canada; 3 Department of Neurosurgery and Neuro-Oncology, 1st Medical Faculty, Central Military Hospital, Charles University, Prague, Czech Republic; 4 Department of Neurosurgery, Landeskrankenhaus Feldkirch, Feldkirch, Austria; 5 Department of Neurosurgery, Medical University Graz, Graz, Austria; 6 Department of Neurosurgery & Neurorestauration, Klinikum Klagenfurt, Klagenfurt, Austria; 7 Department of Neurosurgery, Kepler University Hospital GmbH, Johannes Kepler University, Linz, Austria; 8 Department of Neurosurgery, Christian Doppler Clinic, Paracelsus Medical University, Salzburg, Austria; 9 Department of Neurosurgery, University Hospital of St. Poelten, Karl Landsteiner University of Health Sciences, St. Poelten, Austria; 10 Department of Neurosurgery, Medical University of Vienna, Vienna, Austria; 11 Unit of Neurosurgery, Klinik Landstrasse, Vienna, Austria; 12 Department of Neurosurgery, Klinik Donaustadt, Vienna, Austria; 13 Department of Neurosurgery, General Hospital Wiener Neustadt, Wiener Neustadt, Austria; 14 Department of Neurosurgery, University Hospital Ostrava, Ostrava, Czech Republic; 15 Department of Neurosurgery, Ceske Budejovice Hospital, Ceske Budejovice, Czech Republic; 16 Department of Neurosurgery, Pilsen University Hospital, Pilsen, Czech Republic; 17 Department of Neurosurgery, Motol University Hospital, Prague, Czech Republic; 18 Department of Neurosurgery, University Hospital Hradec Kralove, Hradec Kralove, Czech Republic; 19 Unit of Neurosurgery, Municipal Hospital - Ostrava Fifejdy, Ostrava, Czech Republic; 20 Department of Neurosurgery, University Hospital Kralovske Vinohrady, Prague, Czech Republic; 21 Department of Neurosurgery, University Hospital Olomouc, Olomouc, Czech Republic; 22 Department of Neurosurgery, Usti Nad Labem Hospital, Usti Nad Labem, Czech Republic; 23 Department of Neurosurgery, Liberec Hospital, Liberec, Czech Republic; 24 Unit of Neurosurgery, Homolka Hospital, Prague, Czech Republic; 25 Department of Neurosurgery, University Hospital Brno and Masaryk University, Brno, Czech Republic; 26 Unit of Neurosurgery, Zlin Hospital, Zlin, Czech Republic; 27 Unit of Neurosurgery, Pardubice Hospital, Pardubice, Czech Republic; 28 Department of Neurosurgery, St. Anne’s University Hospital Brno and Masaryk University, Brno, Czech Republic

**Keywords:** Epidemiology, Neurology, Brain injuries, Cerebrovascular disorders, CNS cancer, Hydrocephalus, Spinal cord diseases, Stroke

## Abstract

Reflecting the first wave COVID-19 pandemic in Central Europe (i.e. March 16th–April 15th, 2020) the neurosurgical community witnessed a general diminution in the incidence of emergency neurosurgical cases, which was impelled by a reduced number of traumatic brain injuries (TBI), spine conditions, and chronic subdural hematomas (CSDH). This appeared to be associated with restrictions imposed on mobility within countries but also to possible delayed patient introduction and interdisciplinary medical counseling. In response to one year of COVID-19 experience, also mapping the third wave of COVID-19 in 2021 (i.e. March 16 to April 15, 2021), we aimed to reevaluate the current prevalence and outcomes for emergency non-elective neurosurgical cases in COVID-19-negative patients across Austria and the Czech Republic. The primary analysis was focused on incidence and 30-day mortality in emergency neurosurgical cases compared to four preceding years (2017–2020). A total of 5077 neurosurgical emergency cases were reviewed. The year 2021 compared to the years 2017–2019 was not significantly related to any increased odds of 30 day mortality in Austria or in the Czech Republic. Recently, there was a significant propensity toward increased incidence rates of emergency non-elective neurosurgical cases during the third COVID-19 pandemic wave in Austria, driven by their lower incidence during the first COVID-19 wave in 2020. Selected neurosurgical conditions commonly associated with traumatic etiologies including TBI, and CSDH roughly reverted to similar incidence rates from the previous non-COVID-19 years. Further resisting the major deleterious effects of the continuing COVID-19 pandemic, it is edifying to notice that the neurosurgical community´s demeanor to the recent third pandemic culmination keeps the very high standards of non-elective neurosurgical care alongside with low periprocedural morbidity. This also reflects the current state of health care quality in the Czech Republic and Austria.

## Introduction

Fully unexpected, in March 2020 the pandemic of severe acute respiratory syndrome coronavirus 2 (SARS-CoV-2), known as COVID-19, struck at the heart of human life and has unpredictably changed the world, staying detrimental to all spheres of our daily living. Taking tentative primal epidemiological measures, postponement of elective surgeries was one of many not surprising consecutive yet still rectifiable aftermaths of the global pandemic. However, thenceforwards, alarming data on depleted emergency operations have been announced in various surgical branches^1–4^. Reflecting the peak pandemic of the first wave in the Czech Republic, Austria and Switzerland (i.e. March 16th—April 15th, 2020) the neurosurgical community witnessed a general diminution in the incidence of emergency neurosurgical cases^1^. This was impelled by a reduced number of traumatic brain injuries, spine conditions, and chronic subdural hematomas. Short-term mortality did not significantly differ overall or for any of the conditions investigated during the first wave of the pandemic. Lower incidence of neurosurgical emergency cases appeared to be associated with restrictions imposed on mobility within countries but also to possible delayed patient introduction and interdisciplinary medical counseling^1^.

More than one year after the global pandemic inception (and its worldwide announcement on 11th March 2020), keeping acquainted with the current COVID-19 forms, Central European health care systems have been continuously adjusting their approaches to the present pandemic situation, trying to maximize the quality of care whilst maintaining appropriate epidemiological measures. Meanwhile, in Central Europe the COVID-19 pandemic reached the third peak within the same period as the first wave (i.e. March 16th-April 15th, 2021).

In response to the third wave, we aimed to reevaluate the prevalence and outcomes for neurosurgical emergency cases in COVID-19-negative patients during the matching time period to the first wave (i.e. March 16th—April 15th, 2021) in Austria and the Czech Republic. The primary analysis addressed incidence and 30 day mortality in non-elective neurosurgical cases compared to four preceding years.

## Methods

Mirroring a population of over twenty million people, our analysis comprised data from all neurosurgical centers in Austria and the Czech Republic. In the course of initial outbreak, these countries adjourned scheduled surgeries, and intensive care resources were preserved or redistributed. There was a high endeavor to proceed with non-elective surgeries without any restraints; yet, lacking the official guidelines, it was ambiguous which procedures required instantaneous resolution and what time frames were justifiable to initiate intervention. In defiance of the high prevalence of COVID-19 cases in Austria and the Czech Republic, there was no apparent deficiency in intensive care capacities within the pandemic culminations. Strikingly, the workflow was affected heavily in the first phase of the pandemic with all related uncertainties.

In 2021, similar actions were needed as COVID-19 cases reached a critical threshold. Hence, data on all emergency, cranial and spinal neurosurgical procedures performed during March 16th – April 15th, 2021, as well as 2020 were recorded. This time period was elected to mirror the peaks of the pandemic in these fields over both years. Noteworthy, analogous regulations were applied in both appraised countries over the defined time period. Cases from coincident time periods in the preceding three years (2017 – 2019) were analyzed for reference. Ethical approval was attained by the coordinating national centers (Ethikkommission Nummer der Medizischen Universität Innsbruck 1194/2021 in Austria and Etická komise FN Ostrava 448/2021 in the Czech Republic). All research was performed in conformity with the local guidelines as well as in accordance with the declaration of Helsinki. Informed consent was waived by the approving ethic committees.

### Patients

Demographic parameters such as age, sex and 30 day mortality as well as surgical data were retrospectively recorded in all participating centers. Patients who were treated conservatively, were not enrolled in the study. All inclusion and exclusion criteria are presented in Table 1. Cases were categorized as follows: 1) traumatic brain injury (TBI) requiring any emergent neurosurgical intervention (i.e. monitoring for intracranial pressure, decompressive craniectomy, etc.), 2) chronic subdural hematomas (cSDH) requiring surgical evacuation due to the mass effect and/or neurological symptoms, 3) aneurysmal or non-aneurysmal subarachnoid hemorrhage, and other neurovascular pathologies, including cavernous malformations, arteriovenous fistulae, and arteriovenous malformations necessitating rapid treatment (i.e. latest within 2 weeks after onset), 4) spinal conditions necessitating emergency surgical treatment involving degenerative spine disease with new onset or acute deterioration of neurological symptoms (e.g., cauda equina syndrome and/or motor deficits, or signs of spinal cord compression), spine tumors including metastases, extradural, intradural and intramedullary tumors, infectious spine diseases, as well as traumatic spine injuries with or without neurological deficits, 5) acute deterioration of preexisting hydrocephalus (e.g., shunt dysfunction) or new onset hydrocephalus, and 6) newly diagnosed intracranial pathologies including high- or low-grade gliomas, meningiomas, ischemic or hemorrhagic strokes and intracranial hematomas requiring decompressive craniectomy or emergency surgery, and brain abscesses in need for rapid or emergency surgical scheduling (i.e. latest within 3 weeks after symptom onset or diagnosis).

**Table 1:** Inclusion and exclusion criteria.

Inclusion	Exclusion
Any traumatic brain injury necessitating surgical procedure	Conservative management Elective surgeries Any intervention not meeting the predefined inclusion criteria
Chronic subdural hematoma necessitating surgical treatment	
Subarachnoid hemorrhage and other neurovascular lesions (aneurysms, cavernomas, arteriovenous malformations, dural arteriovenous fistulae,etc.) requiring surgical and/or endovascular treatment	
Any spine lesion (e.g., tumor, traumatic, degenerative, infectious, etc.) requiring emergency surgical treatment	
New-onset or acute deterioration of patients with hydrocephalus	
Intracranial lesions including metastases, high- and low- grade gliomas, meningiomas, strokes, intracranial hematomas, and abscesses requiring emergency surgical treatment	

## Statistics

The number of total incident cases, and number within each category, were determined in each year, separately for Austria and the Czech Republic. Chi-square tests were applied to test for differences in incidence across the years, and significant differences (*P* < 0.05) were further examined using standardized residuals. Rates of 30-day mortality were determined for each year, and within each category separately for Austria and the Czech Republic. Thirty-day mortality was analyzed using logistic regression with year (2021/2020 versus 2017—2019) as an independent variable. All models were adjusted for age (0–18, 18–39, 40–64, 65–74, and 75 + years old) and sex.

## Results

The participating centers in the Czech Republic and Austria recorded 2,242 and 2,835 emergency neurosurgical cases, respectively. Female patients represented 41% of the population in the Czech Republic and 45% in Austria. The median age in both countries was 61 years (ranging between 0 and 97 years in the Czech Republic, and between 0 and 95 years in Austria). One Austrian center could not provide any data but on spine cases for 2019 and 2020, and thus was excluded from descriptions and analyses of total cases and spine cases but was included for the other categories.

### Incidence

Analyses revealed significant differences in incidence rates for cSDH, spine, acute hydrocephalus, and tumor cases in Austria, driven by their lower incidence rates in 2020. Overall incidence of neurosurgical cases demonstrated a significant peak in Austria in 2021. There was no significant difference in the total number of cases in the Czech Republic from 2017–2021. As previously reported, the TBI cases in the Czech Republic were high in 2019, while cSDH cases experience a high in 2018, and a low in 2020, causing significant differences in incidence rates over time. The only novel finding for the Czech Republic was a significant increase in spinal cases in 2021 (Table 2).

**Table 2:** The incidence of emergency neurosurgical cases in Austria and the Czech Republic from March 16th until April 15th 2017–2021.

	Austria					
	2017	2018	2019	2020	2021	P-value*
Total cases N (%)	400	417	392	345	**460**	0.002*
**Condition**						
TBI	42 (10.5%)	43 (10.3%)	26 (6.6%)	27 (7.8%)	34 (7.4%)	0.11
cSDH	72 (18.0%)	63 (15.1%)	62 (15.8%)	**38** (**11.0%**)	72 (15.7%)	0.01*
SAH and Other Vascular Lesions	26 (6.5%)	47 (11.3%)	37 (9.4%)	41 (11.9%)	46 (10.0%)	0.12
Spine Lesions	104 (26.0%)	110 (26.4%)	111 (28.3%)	**71** (**20.6%**)	117 (25.4%)	0.01*
Acute Hydrocephalus	59 (14.8%)	42 (10.1%)	58 (14.8%)	**28** (**8.1%**)	72 (15.7%)	< 0.001*
Tumor and Other Intracranial Lesions	105 (26.2%)	126 (30.2%)	105 (26.8%)	**152** (**44.1%**)	130 (28.3%)	0.01*

	Czech Republic					
	2017	2018	2019	2020	2021	P-value*
Total cases N (%)	586	551	584	513	601	0.07
**Condition**						
TBI	74 (12.6%)	68 (12.3%)	**98** (**16.8%**)	48 (9.4%)	62 (10.3%)	< 0.001*
cSDH	87 (14.8%)	**110** (**20.0%**)	84 (14.4%)	**53** (**10.3%**)	72 (12.0%)	< 0.001*
SAH and Other Vascular Lesions	59 (10.1%)	59 (10.7%)	66 (11.3%)	63 (12.3%)	59 (9.8%)	0.96
Spine Lesions	175 (29.9%)	139 (25.2%)	135 (23.1%)	153 (29.8%)	**208** (**34.6%**)	< 0.001*
Acute Hydrocephalus	53 (9.0%)	42 (7.6%)	59 (10.1%)	45 (8.8%)	42 (7.0%)	0.32
Tumor and Other Intracranial Lesions	138 (23.5%)	133 (24.1%)	142 (24.3%)	151 (29.4%)	158 (26.3%)	0.59

*TBI* Traumatic brain injury, *cSDH* chronic subdural hematoma, *SAH* subarachnoid hemorrhage and other vascular lesions;

*P-values derived from chi-square tests.

Significant values are in [bold].

### Demographics

#### Traumatic brain injury

There were no differences in patients´ age, TBI grade or initial GCS between the years in both Austria and the Czech Republic. The details are listed in Tables 3 and 4.

**Table 3:** The demographics related to all analyzed conditions in Austria.

Austria						
		2017	2018	2019	2020	2021
**TBI**						
	Age (median, IQR)	57.0 (41.0–73.0)	52.0 (36.0–71.0)	57.5 (35.0–71.5)	60.0 (38.0–74.5)	48.5 (34.25–61.0)
	Sex (male)	27 (64.3%)	29 (69.0%)	18 (69.2%)	17 (63.0%)	22 (64.7%)
	TBI grade	2.0 (1.0–3.0)	2.0 (1.0–3.0)	3.0 (1.0–3.0)	2.0 (1.0–3.0)	2.5 (2.0–3.0)
	Initial GCS	10.0 (6.5–13.25)	9.0 (6.0–14.0)	7.0 (5.75–14.25)	12.0 (8.0–13.75)	8.0 (3.0–13.25)
	GOSE (median, IQR)	4.0 (3.0–7.0)	3.5 (3.0–6.75)	4.0 (3.0–6.0)	4.0 (3.0–7.0)	4.0 (3.0–6.0)
**cSDH**						
	Age	74.0 (65.6–73.0)	77.0 (65.5–83.5)	76.0 (69.25–81.0)	76.0 (66.25–81.75)	76.5 (67.0–81.25)
	Sex (male)	47 (65.3%)	37 (58.7%)	43 (69.4%)	27 (71.1%)	52 (72.2%)
	Initial GCS	15.0 (15.0–15.0)	15.0 (14.0–15.0)	15.0 (15.0–15.0)	15.0 (14.0–15.0)	15.0 (14.0–15.0)
	GOSE	8.0 (6.0–8.0)	7.0 (6.0–8.0)	8.0 (6.0–8.0)	7.0 (5.0–8.0)	7.0 (6.0–8.0)
**SAH and Other Vascular Lesions**						
	Age	58.2 (53.5–66.63)	59.5 (50.75–68.25)	55.0 (51.0–61.0)	61.48 (46.3–70.0)	58.0 (46.0–68.0)
	Sex (male)	9 (33.3%)	24 (46.2%)	19 (44.2%)	21 (46.7%)	12 (27.9%)
	Initial GCS	15.0 (13.0–15.0)	14.5 (10.75–15.0)	14.5 (10.5–15.0)	14.0 (12.0–15.0)	12.5 (3.0–15.0)
	Aneurysms N (%)	24 (88.9%)	45 (88.2%)	36 (83.7%)	32 (72.7%)	35 (83.3%)
	Hunt&Hess Classification	2.0 (1.0–3.0)	2.0 (1.0–3.0)	2.0 (1.0–4.0)	2.0 (1.0–4.0)	2.0 (2.0–4.0)
	Fisher Classification	3.0 (2.0–4.0)	2.5 (2.0–4.0)	2.5 (1.0–4.0)	4.0 (2.0–4.0)	4.0 (1.0–4.0)
	Last mRS	3.0 (1.0–4.0)	3.0 (1.0–4.0)	1.0 (0.0–3.0)	2.5 (1.0–5.0)	2.0 (1.0–4.0)
	Timing of Aneurysm Treatment	2.0 (1.0–2.0)	1.0 (1.0–2.0)	1.0 (1.0–2.25)	1.0 (0.0–2.0)	1.0 (1.0–1.0)
	GOSE	5.0 (3.0–7.0)	5.0 (3.0–7.25)	7.0 (2.75–8.0)	5.5 (3.0–7.0)	1.0 (0.0–5.0)
**Spine Lesions**						
	Age	59.0 (48.25–69.0)	54.5 (43.25–64.0)	60.0 (46.0–70.0)	53.0 (41.0–69.0)	60.0 (47.0–72.25)
	Sex (male)	54 (51.9%)	64 (58.2%)	63 (56.8%)	40 (56.3%)	73 (60.8%)
	Timing from Symptoms to Surgery	3.0 (3.0–4.0)	4.0 (3.0–5.0)	4.0 (2.5–5.0)	3.0 (3.0–4.5)	4.0 (3.0–5.0)
	GOSE	7.0 (6.0–8.0)	7.5 (7.0–8.0)	7.0 (6.0–7.0)	7.0 (6.0–8.0)	7.0 (6.0–7.0)
**Acute Hydrocephalus**						
	Age	54.0 (24.0–68.5)	54.5 (35.0–76.75)	53.0 (10.25–69.0)	42.0 (13.0–56.0)	59.0 (49.5–67.25)
	Sex (male)	36 (61.0%)	24 (57.1%)	28 (48.3%)	13 (48.1%)	34 (47.2%)
	New Onset of Acute Hydrocephalus	32 (54.2%)	22 (52.4%)	28 (48.3%)		48 (66.7)
	GOSE	7.0 (4.0–8.0)	7.0 (4.0–8.0)	6.0 (3.0–8.0)	6.5 (4.0–8.0)	4.0 (3.0–6.75)
**Tumors and Other Intracranial Lesions**						
	Age	62.0 (48.0–71.0)	64.0 (53.0–73.0)	59.0 (48.0–70.25)	62.0 (54.0–70.0)	62.0 (51.0–72.0)
	Sex (male)	55 (53.4%)	68 (54.0%)	58 (55.8%)	76 (51.0%)	59 (45.7%)
	Seizures	9 (8.7%)	20 (16.0%)	23 (22.1%)	21 (14.3%)	25 (19.4%)
	Any Focal Neurological Deficit	73 (70.9%)	86 (68.3%)	70 (67.3%)	100 (68.5%)	90 (69.8%)
	GOSE	6.0 (4.0–8.0)	6.0 (4.0–7.0)	6.0 (3.0–7.0)	6.0 (4.0–7.0)	6.0 (4.0–7.25)

*TBI* Traumatic brain injury, *cSDH* chronic subdural hematoma, *SAH* subarachnoid hemorrhage and other vascular pathologies; *GOSE* Glasgow Outcome Scale-Extended at discharge.

**Table 4:** The demographics related to all analyzed conditions in the Czech Republic.

Czech Republic						
		2017	2018	2019	2020	2021
**TBI**						
	Age (median, IQR)	57.5 (43–71.75)	63.0 (39.5–74.0)	64.5 (39.25–75.0)	62.5 (40.75–71.75)	59.0 (43.0–68.75)
	Sex (male)	48 (64.9%)	51 (75.0%)	69 (70.4%)	36 (75.0%)	44 (71.0%)
	TBI grade	3.0 (2.0–3.0)	3.0 (1.0–3.0)	3.0 (1.0–3.0)	3.0 (1.0–3.0)	2.0 (1.0–3.0)
	Initial GCS	8.0 (3.0–13.75)	5.0 (3.0–13.0)	6.0 (3.0–13.0)	6.5 (3.0–12.0)	9.5 (4.25–14.0)
	GOSE (median, IQR)	4.0 (2.0–6.0)	3.0 (2.0–5.0)	3.0 (2.0–5.0)	3.0 (2.0–6.0)	3.5 (2.0–6.0)
**cSDH**						
	Age	73.0 (62.5–79.5)	71.5 (63.25–80.75)	71.5 (64.0–77.25)	75.0 (71.0–81.0)	75.0 (69.0–80.0)
	Sex (male)	70 (80.5%)	84 (76.4%)	57 (67.9%)	37 (69.8%)	45 (61.6%)
	Initial GCS	15.0 (14.0–15.0)	15.0 (14.0–15.0)	15.0 (14.0–15.0)	15.0 (14.0–15.0)	15.0 (14.0–15.0)
	GOSE	7.0 (5.0–7.5)	7.0 (5.0–8.0)	7.0 (5.0–8.0)	7.0 (4.0–8.0)	7.0 (6.0–7.25)
**SAH and Other Vascular Lesions**						
	Age	60.0 (50.5–68.5)	60.0 (51.0–67.5)	64.0 (51.0–71.0)	62.0 (48.5–71.0)	58.0 (47.5–69.5)
	Sex (male)	28 (47.5%)	32 (54.2%)	29 (43.9%)	28 (44.4%)	34 (58.6%)
	Initial GCS	14.0 (11.0–15.0)	14.0 (6.5–15.0)	14.5 (10.0–15.0)	14.0 (7.5–15.0)	15.0 (12.0–15.0)
	Aneurysms N (%)	21 (39.6%)	34 (50%)	33 (55.9%)	31 (52.5%)	24 (40.7%)
	Hunt&Hess Classification	1.0 (0.0–3.25)	2.0 (0.0–3.0)	2.0 (0.0–3.0)	2.0 (1.0–4.0)	1.0 (0.0–3.0)
	Fisher Classification	2.0 (0.0–4.0)	3.0 (0.0–4.0)	3.0 (0.0–4.0)	3.0 (0.0–4.0)	3.0 (1.0–4.0)
	Last mRS	2.0 (1.0–5.0)	2.0 (0.0–5.0)	2.0 (1.0–5.0)	3.0 (1.0–5.0)	2.0 (1.0–3.0)
	Timing of Aneurysm Treatment	2.0 (1.0–2.0)	1.0 (1.0–2.0)	2.0 (2.0–4.0)	1.0 (1.0–3.0)	1.0 (1.0–1.0)
	GOSE	6.0 (3.0–7.5)	6.0 (3.0–7.0)	5.0 (3.0–7.0)	4.0 (3.0–7.0)	6.0 (4.5–7.0)
**Spine Lesions**						
	Age	58.0 (45–68)	57.0 (44.0–70.0)	62.0 (42.5–71.5)	55.0 (44.0–69.0)	58.4 (45.0–69.25)
	Sex (male)	105 (60.0%)	93 (66.9%)	87 (64.4%)	104 (68.0%)	124 (59.6%)
	Timing from Symptoms to Surgery	3.0 (1.0–4.0)	3.0 (1.0–4.0)	3.0 (1.5–4.0)	3.0 (1.0–4.0)	4.0 (2.0–5.0)
	GOSE	7.0 (6.0–8.0)	6.0 (5.0–7.0)	6.0 (4.0–7.0)	6.5 (5.0–7.0)	7.0 (5.75–8.0)
**Acute Hydrocephalus**						
	Age	17.0 (1.0–55.0)	41.0 (7.25–67.0)	47.0 (3.5–64.5)	56.0 (17.0–70.0)	39.0 (15.0–81.0)
	Sex (male)	32 (60.4%)	19 (45.2%)	23 (39.0%)	20 (44.4%)	25 (59.5%)
	New Onset of Acute Hydrocephalus	29 (54.7%)	25 (59.5%)	34 (57.6%)	31 (68.9%)	24 (57.1%)
	GOSE	6.0 (4.0–7.0)	6.0 (4.0–8.0)	5.0 (3.0–7.0)	5.0 (3.0–7.0)	6.0 (5.0–8.0)
**Tumors and Other Intracranial Lesions**						
	Age	58.5 (41.25–70.75)	55.0 (43.0–68.0)	61.0 (48.75–70.0)	59.0 (46.0–70.0)	61.0 (46.0–71.75)
	Sex (male)	72 (52.2)	68 (51.1%)	69 (48.6%)	67 (44.4%)	64 (40.5%)
	Seizures	23 (16.7%)	21 (15.8%)	23 (16.2%)	28 (18.5%)	24 (15.2%)
	Any Focal Neurological Deficit	96 (70.6%)	98 (73.7%)	99 (69.7%)	101 (66.9%)	111 (70.3%)
	GOSE	6.0 (4.0–7.0)	6.0 (4.0–7.0)	5.0 (3.0–7.0)	6.0 (5.0–7.0)	6.0 (5.0–7.0)

*TBI* Traumatic brain injury, *cSDH* chronic subdural hematoma, *SAH* subarachnoid hemorrhage and other vascular pathologies; *GOSE* Glasgow Outcome Scale-Extended at discharge.

#### Chronic subdural hematoma

There were no differences in patients´ age or initial GCS between the years in both Austria and the Czech Republic. The results are listed in Tables 3 and 4.

#### Subarachnoid hemorrhage and other neurovascular lesions

There were no differences in patients´ age, SAH Hunt & Hess classification grades or Fisher score grades between the years in both Austria and the Czech Republic. Timing of treatment did not differ across the years in both countries. The results are listed in Tables 3 and 4.

#### Spine lesions

There were no differences in patients´ age between the years in both Austria and the Czech Republic. Timing from related symptoms to surgery did not differ across the years in both countries. The results are listed in Tables 3 and 4.

#### Acute hydrocephalus

Mean age of patients varied between the years in both Austria and the Czech Republic. New onset of acute hydrocephalus ranged between 44.4% and 66.7% in Austria and from 54.7% to 68.9% in the Czech Republic, with the highest rates occurring either in 2020 or in 2021. The results are listed in Tables 3 and 4.

#### Tumors and other intracranial lesions

There were no differences in patients´ age between the years in both Austria and the Czech Republic. Related symptoms ranged between 67.3 and 70.9% of patients across the years in Austria and from 66.9 to 73.7% of patients in the Czech Republic. The results are listed in Tables 3 and 4.

### Thirty day mortality

Of the collected cases in the Czech Republic and Austria, 2314 (> 99.9% of total) in the Czech Republic, and 2220 (99.0%) in Austria, were included in the logistic regression analysis of short-term mortality. Logistic regression models imparted that the year 2021, compared to the years 2017–2019, did not significantly correlate with any increased odds of 30-day mortality in Austria or in the Czech Republic (Tables 5 and 6).

**Table 5:** The rates of 30-day mortality from emergency neurosurgical cases in Austria and the Czech Republic from 2017–2021.

Austria					
All conditions	2017	2018	2019	2020	2021
	6.0%	6.0%	3.6%	6.5%	3.5%
**Condition**					
TBI	12.2%	14.3%	3.8%	7.7%	9.1%
cSDH	2.9%	1.6%	0.0%	5.3%	2.8%
SAH and Other Vascular Lesions	8.0%	13.3%	16.2%	9.8%	13.6%
Spine Lesions	0.0%	0.9%	0.0%	1.4%	0.0%
Acute Hydrocephalus	8.5%	0.0%	3.4%	7.4%	1.4%
Tumor and Other Intracranial Lesions	9.7%	8.7%	6.7%	8.7%	3.9%

Czech Republic					
All conditions	2017	2018	2019	2020	2021
	5.1%	7.1%	8.4%	4.5%	5.5%
**Condition**					
TBI	13.5%	20.9%	17.3%	4.3%	21.0%
cSDH	2.3%	4.5%	6.0%	5.9%	5.6%
SAH	15.3%	5.1%	6.1%	10.0%	5.1%
Spine	1.1%	2.9%	3.7%	1.3%	1.9%
Acute hydrocephalus	5.7%	9.5%	8.5%	6.7%	4.9%
Tumor and other intracranial lesions	2.9%	6.8%	9.3%	4.6%	4.4%

*TBI* Traumatic brain injury, *cSDH* chronic subdural hematoma, *SAH* subarachnoid hemorrhage and other vascular lesions.

**Table 6:** Logistic regression of 30-day mortality in emergency neurosurgical cases in Austria and the Czech Republic. Effects estimates reflection 2021 compared to aggregate of reference years (2017–2019).

Country	Condition	Effect (95% CI)	*P*-value*
Austria	All	0.67 (0.37–1.14)	0.16
	TBI	0.98 (0.20–3.62)	0.98
	cSDH	3.02 (0.34–26.8)	0.29
	SAH and Other Vascular Lesions	0.92 (0.27–2.70)	0.89
	Spine Lesions	0.00 (NA)	1.00
	Acute Hydrocephalus	0.32 (0.02–1.96)	0.30
	Tumor and Other Intracranial Lesions	0.44 (0.14–1.09)	0.10
Czech Republic	All	0.79 (0.52–1.16)	0.24
	TBI	1.40 (0.65–2.94)	0.38
	cSDH	1.28 (0.34–3.90)	0.69
	SAH and Other Vascular Lesions	0.55 (0.12–1.79)	0.37
	Spine Lesions	0.74 (0.20–2.26)	0.62
	Acute Hydrocephalus	0.54 (0.08–2.21)	0.45
	Tumor and Other Intracranial Lesions	0.63 (0.24–1.42)	0.29

*CI* confidence interval, *TBI* Traumatic brain injury, *cSDH* chronic subdural hematoma, *SAH* subarachnoid hemorrhage and other vascular pathologies.

*All models adjusted for age and sex.

### Neurological outcome at discharge

#### Traumatic brain injury

There were no differences in GOSE score across the years in both Austria and the Czech Republic with a mean grade ranging between 3 and 4 in both countries. The results are listed in Tables 3 and 4.

#### Chronic subdural hematoma

GOSE score did not differ across the years in both Austria and the Czech Republic, with a mean grade of 7 or 8 in Austria and an annual mean grade of 7 in the Czech Republic. The results are listed in Tables 3 and 4.

#### Subarachnoid hemorrhage and other neurovascular lesions

Both mean mRS and GOSE scores varied across the years in Austria ranging between 1 and 3, and from 1 to 7, respectively. In the Czech Republic, there were no differences in mRS score, with an annual mean grade of 2 except for 2020 where the mean grade was 3. GOSE score ranged between the mean grades 4 and 6 across the years. The results are listed in Tables 3 and 4.

#### Spine lesions

There were no differences in GOSE score across the years in both Austria and the Czech Republic, with an annual mean grade of 7 in Austria and a mean grade ranging from 6 to 7 in the Czech Republic. The results are listed in Tables 3 and 4.

#### Acute hydrocephalus

GOSE score varied across the years in Austria, with a mean grade ranging from 4 to 7, and the lowest grade in 2021. There were no differences in GOSE score across the years in the Czech Republic, with a mean grade of 5 or 6. The results are listed in Tables 1 and 2.

#### Tumors and other intracranial lesions

There were no differences in GOSE score across the years in both Austria and the Czech Republic, with an annual mean grade of 6 in both countries, except for 2019 in the Czech Republic where the mean grade was 5. The results are listed in Tables 3 and 4.

## Discussion

Our current study reflecting the present status of health care services in the Czech Republic and in Austria imparted an overall significant propensity toward increased incidence rates of non-elective, emergency neurosurgical cases during the recent third COVID-19 pandemic wave in Austria, driven by their lower incidence during the first COVID-19 culmination in 2020. This actuality is confirmed partly also in the Czech Republic, markedly in spine cases. Selected neurosurgical conditions commonly associated with traumatic etiologies including TBI, and chronic subdural hematomas roughly reverted to similar incidence rates from the previous non-COVID-19 years. In defiance of long-suffering health care services and intensive care, the prior lower prevalence of spine cases and acute hydrocephalus in Austria also normalized. Again, albeit varying across the study period (2017–2021), 30-day mortality during the continuing COVID-19 pandemic in Austria and the Czech Republic was not substantially altered overall or for any condition compared to previous years. Mirroring the current state of health care quality in both Central European countries, our data anew indicate that emergency neurosurgical care continues to be provided at high standard level regardless of the COVID-19 pandemic.

The COVID-19 pandemic has initiated unparalleled barriers to the delivery of health care all around the world^5,6^. In general, surgical practices have been substantially afflicted by the persisting COVID-19 pandemic. Above all, the most inauspicious impact experienced the preventive health care, with innumerable (infinite) postponed elective surgeries in every surgical field^5–11^. Urgently reacting to the first pandemic culmination, several national guidelines devoted to elective case scheduling during a pandemic were announced by national authorities, for instance, the American College of Surgeons (ACS)^12^ and the CMS^12,13^. Some protocols accentuated social distancing as an imperative measure to curtail viral spread^14,15^. Thereafter, a series of federal propositions and administrative regulations from 31 states asserted all intended surgical interventions and elective inpatient diagnostic examinations to be postponed^16–19^. However, the dichotomization of elective versus non-elective interventions was condemned for incompetently risk-stratifying patients^20^. The potential adverse effects consequent upon a delay in a scheduled intervention differ by neurosurgical specialty, whether neurooncology, vascular, functional, pediatrics, or spine^21^.

The collective Congress of Neurological Surgery and American Association of Neurological Surgeons (CNS/AANS) Tumor Sect. ^22^ declared their own protocol given the unique nature of neurooncological diseases. They urged surgical interventions for all newly diagnosed cranial or spinal metastatic diseases, and high-grade gliomas. They were exponents of narrow follow-ups for benign asymptomatic pathologies and low-grade gliomas. The CMS^13^ propounded a three-level algorithm wherein low-acuity services were rescheduled or effected via telemedicine, intermediate-acuity services were triaged, and high-acuity were pursued without any significant delay. Similarly, the European Association of Neurological Societies (EANS)^23^ advised triages of neurosurgical interventions based on a categorization of emergency conditions. This tool was based on the Elective Surgery Acuity Scale (ESAS)^24^ from St. Louis University. While the tool gives examples of neurosurgical procedures in each category, the EANS exhorted the national neurosurgical societies to create more granular triage schemes based on regional capacity.

Dealing with non-elective emergency cases, for instance, the German Association of Neurological Surgeons (Deutsche Gesellschaft für Neurochirurgie – DGNC)^25^ introduced general guidelines stating that risks of death as well as of the permanent morbidity owing to surgery postponement in these patients must be weighed against the risks of COVID-19 disease. Including all relevant neurosurgical fields, neurosurgical patients who might be threatened by deferral of indicated neurosurgical treatment are to be treated as an emergency irrespective of current COVID-19 restrictions. Our present data are in harmony with these recommendations. Standing the rough continuing COVID-19 pandemic situation, the Austrian and Czech neurosurgical community have been succeeding in keeping high standards of emergency neurosurgical health services, without any pertinent threats for our patient population. Regrettably, this is not a matter of course in all other health care spheres^26^. Ultimately, one should also keep in mind that there might be more indirect erratic repercussions in the future concerning not only neurosurgical patients, for example the resulting lack of neurosurgical training for our residents. All these possible implications will require appropriate handling of the topical situation.

Health care services worldwide continue to improvise, ceaselessly revising and optimizing the current local protocols to maintain possibly the most effective level of performance amidst substantial scarcity in equipment and facilities. Being in a lucky position in social welfare Central Europe, quality of care remains at the high level, not yet directly troubled with handling COVID-19 complications.

It is worth of noting that, for instance, in Austria during the very first pandemic culmination the occupancy of ICU beds reserved for COVID patients ranged between 5 and 18%. In the current study period, the ICU beds occupancy substantially increased, ranging from 55 to 68% by the same number of 682 reserved ICU beds (https://covid19.healthdata.org/austria?view=resource-use&tab=trend&resource=all_resources). The similar trend occurred also in the Czech Republic (https://onemocneni-aktualne.mzcr.cz/vyvoj-kapacit-luzkove-pece). Specifically, in April 2020, there were on average 55 ventilated patients with COVID-19 whereas one year later (April 2021) there were 531 such patients (https://onemocneni-aktualne.mzcr.cz/api/v2/covid-19). Taking heed of constitutive COVID-19 measures during the first and third pandemic waves, most principal restrictions such as general shutdown, isolation of COVID patients etc. remained similar during both time periods, as in the hospitals so for the public. Importantly, given the one-year intense experience and the resultant acquired skills with treatment of COVID-19 patients, all related procedures became routine in daily clinical practice regardless of substantially increased number of intensive care patients. Also, access to all necessary consumables improved with time.

Importantly, in defiance of this evident growing need of ICU beds, we did not observe any germane effect on the provided neurosurgical care in both countries. By contrast, there was an overall significant propensity toward increased incidence rates of non-elective, emergency neurosurgical cases during the recent third COVID-19 pandemic wave in 2021. Up to now, strictly following our neurosurgical knowledge and contemporary standard operating procedures in both countries, our data revealed no worsening in any of patient outcomes in all examined fields. Unlike, Patel et al.^21^ observed trends of deterioration in neurological findings, malignant dissemination, abscesses in non-traumatic spine cases, and the progress of structural instability in the first wave of the pandemic.

As both countries Austria and the Czech Republic keep adapting to the COVID-19 situation, a better cognizance of the inordinate repercussions on definite subspecialties can acquaint targeted care reescalation in all health care programs and future research appraising the ramifications of pandemic on long-term outcome. Within neurosurgical practice, a detailed analysis of all pertinent effects of the COVID-19 pandemic alongside with the current measures is instrumental in refining the necessary requirements and protocols. Sharing the same opinion^21^, for future contagions or akin health catastrophes, validated predictive models shall notify an outcome-based framework to better delineate scheduled surgery and the straight surgical care of patients, allocating all necessary surgical resources.

Leading strengths of our study were the large sample size alongside with inclusion of multiple reference periods (2017–2019) for comparison to the first COVID-19 culmination in 2020 and the recent pandemic wave in the same time period in 2021 (i.e. March 16th–April 15th) and representation of all neurosurgical centers in Austria and the Czech Republic. Yet, the interpretation of our data is bounded by the retrospective and observational nature of the study. Also, only patients who were treated surgically in an emergency fashion, were analyzed. Hence, surgical prioritization during the proceedings of the COVID-19 pandemic as well as thenceforth persists fundamental^27^. Ultimately, one shall be aware that COVID-19 pandemic exerts deleterious influence upon diverse diseases, leading also to consequential neurological manifestations^28,29^ and some patients may require neurosurgical care.

## Conclusion

Further resisting the major deleterious effects of the continuing global COVID-19 pandemic, it is, again, edifying to notice that the neurosurgical community´s demeanor to the recent another pandemic culmination in at least Central Europe keeps the very high standards of non-elective neurosurgical care alongside with low periprocedural morbidity. This also reflects the current state of health care quality in both countries Austria and the Czech Republic. Yet, one may not rest on their own laurels.
